# Comparison of surgical outcomes of slanted procedure for exotropia with convergence insufficiency according to their response to preoperative monocular occlusion

**DOI:** 10.1038/s41598-020-64251-6

**Published:** 2020-04-29

**Authors:** Bo Young Chun, Jun Ho Oh, Hyung Jun Choi

**Affiliations:** 1 Department of Ophthalmology, School of Medicine, Kyungpook National University, Daegu, Korea; 2 Brain Science & Engineering Institute, School of Medicine, Kyungpook National University, Daegu, Korea

**Keywords:** Diseases, Medical research

## Abstract

The aim of this prospective study was to compare surgical outcomes of slanted bilateral lateral rectus (LR) recession for intermittent exotropia (IXT) with convergence insufficiency (CI) according to their response to preoperative monocular occlusion. This prospective study included 55 children who underwent slanted bilateral LR recession for IXT with CI. Patients were divided into two groups according to their response to preoperative monocular occlusion for 2 hours. The True CI group was defined as having near-distance differences of ≥10 PD before and after occlusion; the Masked CI group as having near-distance differences of <10 PD and ≥10 PD prior to and after occlusion. Slanted procedure reduced distance and near exodeviations from 32.1 PD and 43.0 PD to 3.5 PD and 4.4 PD, and collapsed near-distance differences from 10.9 PD to 1.0 PD at 3 years postoperatively. Cumulative probabilities of surgical success were 76%, and the mean recurrence was 50 months at 3 years postoperatively; the True CI and Masked CI groups showed cumulative success rates of 89% and 55%, respectively (p = 0.0052). Patients in the True CI group demonstrated surgical outcomes superior to those demonstrated by patients in the Masked CI group after slanted bilateral LR recession.

## Introduction

Intermittent exotropia (IXT) with convergence insufficiency (CI) is defined as having a greater exodeviation at near than at distance by ≥10 prism diopters (PD), which occurs in only 1.2–7.8% of IXT cases^1–4^. The etiology of IXT with CI remains unclear, but it may be associated with a weak fusional convergence, a low accommodative convergence to accommodation (AC/A) ratio, tenacious distant fusional drive, or reduced accommodative amplitudes^1,5^. Nonsurgical treatments can relieve symptoms related to IXT with CI, including headache, diplopia, blurred vision, and reading problems^5,6^. However, strabismus surgery is often required to prevent loss of binocular vision in patients who do not respond to nonsurgical treatments, or have a considerably large exodeviation^6–8^. Various surgical procedures for IXT with CI have been introduced in addition to the conventional recession or resection, including the use of adjustable sutures, slanted lateral rectus (LR) recession, slanted medial rectus (MR) resection, and unilateral surgery biased toward MR strengthening than toward LR weakening^1,5,7–16^. However, most of the procedures resulted in unsatisfactory success rates between 18% and 67%, and some patients often experienced limitation of abduction or postoperative diplopia^10,12,17,18^.

Snir *et al*.^1^ introduced slanted LR recession, which demonstrated the best success rate of 92% in 12 patients until 12 months after surgery. This surgical technique recesses the lower fibers of the LR muscle more than the upper fibers; thus, it improves postoperative alignments and collapses near-distance differences in IXT with CI^1^. However, most patients in their study were adults, and other studies on slanted LR recession had a small sample size and short follow-up periods^15,16^. Additionally, some patients with basic type IXT may demonstrate a greater exodeviation at near fixation after prolonged monocular occlusion, thereby resulting in IXT with CI^13^. Therefore, surgical outcomes of slanted LR recession for IXT with CI might differ in their response to monocular occlusion prior to surgery. The present study investigated the long-term results of slanted bilateral LR recession for IXT with CI in children. In addition, we divided these children into two groups according to their response to preoperative monocular occlusion for 2 hours, and compared surgical outcomes between the two groups.

## Results

This prospective study included a total of 55 patients, with 35 (63.6%) patients in the True CI group and 20 (36.4%) patients in the Masked CI group. Preoperative patient demographics were not significantly different between the two groups (Table 1).

**Table 1:** Preoperative demographics of all subjects in this study as well as the two groups of intermittent exotropia with convergence insufficiency according to their response to preoperative monocular occlusion for 2 hours.

	All subjects	True CI Group	Masked CI Group	P value
Number of patients	55	35	20	
Age at surgery (years) (mean ± SD)	9.2 ± 6.2	9.0 ± 6.3	9.3 ± 5.8	0.863*
Sex, number (%)				0.786^†^
Male	26 (47%)	19	10	
Female	29 (53%)	16	10	
**Preoperative deviation (PD)**				
Distance	32.1 ± 4.4	32.4 ± 4.3	31.5 ± 4.6	0.454*
Near	43.0 ± 4.7	43.7 ± 4.6	41.8 ± 4.7	0.135*
Near-Distance difference	10.9 ± 2.2	11.3 ± 2.5	10.3 ± 1.1	0.09*
Last follow-up (months) (mean ± SD)	53.1 ± 12.5	56.9 ± 6.1	52.9 ± 5.1	0.137*

CI = convergence insufficiency; Masked CI group = near-distance differences of <10 PD before monocular occlusion and ≥10 PD after diagnostic occlusion; PD = prism diopters; Recurrence = recurrence of exotropia of >8 PD; SD = standard deviation; True CI group = near-distance difference of ≥10 PD before and after diagnostic occlusion;

^*^P values calculated using Mann-Whitney U test between True CI and Masked CI groups.

^†^P value calculated using Fisher’s exact test between True CI and Masked CI groups.

The study group showed a significant reduction in the means of both distance and near exodeviations after surgery (Table 2). The preoperative mean exodeviation was 32.1 ± 4.4 PD at distance and 43.0 ± 4.7 PD at near. At 3 years after performing slanted bilateral LR recession, the mean exodeviation was reduced to 3.5 ± 4.7 PD at distance and to 4.4 ± 5.9 PD at near. No differences were found in preoperative mean exodeviations and most of postoperative mean exodeviations between the two groups. However, at 3 years after surgery, the Masked CI group had a significantly greater mean exodeviation at near (6.7 ± 6.8 PD) than the True CI group (3.3 ± 5.1 PD) (p = 0.041).

**Table 2:** Changes in postoperative mean deviations at distance and at near fixation, and the mean amount of near-distance differences over time. (PD ± mean SD).

	All subjects	True CI Group	Masked CI Group	P value*
**Postop 6 months**				
Distance	1.8 ± 3.7	1.4 ± 2.9	1.5 ± 7.9	0.93
Near	3.2 ± 4.9	2.5 ± 4.3	2.9 ± 9.5	0.84
Near-Distance difference	1.4 ± 3.3	1.1 ± 3.3	1.9 ± 3.2	0.41
**Postop 12 months**				
Distance	2.6 ± 3.5	2.2 ± 3.3	3.5 ± 3.9	0.22
Near	3.5 ± 4.7	2.9 ± 4.3	4.8 ± 5.3	0.14
Near-Distance difference	0.9 ± 3.2	0.6 ± 2.6	1.4 ± 4.3	0.43
**Postop 24 months**				
Distance	3.2 ± 5.2	2.4 ± 4.5	4.8 ± 6.2	0.09
Near	4.3 ± 6.0	3.3 ± 4.9	6.4 ± 7.3	0.06
Near-Distance difference	1.1 ± 2.8	0.9 ± 2.7	1.5 ± 3.0	0.41
**Postop 36 months**				
Distance	3.5 ± 4.7	2.9 ± 4.5	4.7 ± 5.1	0.17
Near	4.4 ± 5.9	3.3 ± 5.1	6.7 ± 6.8	0.041
Near-Distance difference	1.0 ± 3.4	0.4 ± 2.9	2.0 ± 4.1	0.12

CI = convergence insufficiency; Masked CI group = near-distance differences of <10 PD before diagnostic occlusion and ≥10 PD after diagnostic occlusion; PD  = prism diopters; Postop = postoperative; SD = standard deviation; True CI group = near-distance difference ≥10 PD before and after diagnostic occlusion;

*P value calculated using Mann-Whitney U test between True CI and Masked CI groups.

At 3 years after surgery, the overall mean near-distance differences successfully collapsed from 10.9 ± 2.2 PD to 1.0 ± 3.4 PD (p < 0.001) as well as near-distance differences of the True CI (11.3 ± 2.5 PD to 0.4 ± 2.9 PD, p < 0.001) and Masked CI (10.3 ± 1.1 PD to 2.0 ± 4.1 PD, p = 0.001) groups. However, there was no difference in the postoperative mean near-distance differences between the True CI and Masked CI groups at 3 years after surgery (p = 0.12). Each millimeter of the slanted LR recession was associated with an 8.8 PD improvement in the near-distance difference. The impact of slanted LR recession on the collapse of near-distance differences (9.8 PD/mm) in the True CI group was significantly superior to a 7.2 PD/mm collapse in the Masked CI group (p = 0.027).

At 3 years after surgery, the mean stereopsis significantly increased from 460 arcsec preoperatively to 150 arcsec in all subjects. (p = 0.03) Improved stereopsis was observed in 17 (48.6%) of 35 and 10 (50%) of 20 patients of the True CI and Masked CI groups, respectively. No change in stereopsis was observed in 18 (51.4%) of 35 and 9 (45%) of 20 patients, respectively, whereas decreased stereopsis was observed in 0 (0%) of 35 and 1 (5%) of 20 patients, respectively, these findings were not significantly different between the two groups. (p = 0.073) (Table 3).

**Table 3:** Changes in stereopsis between True CI and Masked CI groups at 3 years after slanted lateral rectus recession.

	Improved	No Change	Decreased	p-value*
True CI group	17 (48.6%)	18 (51.4%)	0 (0%)	0.073
Masked CI group	10 (50%)	9 (45%)	1 (5%)	

CI  = convergence insufficiency; Masked CI group = near-distance differences of <10 PD before diagnostic occlusion and ≥10 PD after diagnostic occlusion; True CI group = near-distance difference ≥10 PD before and after diagnostic occlusion;

*P value calculated using Fisher’s exact test between True CI and Masked CI groups.

Surgical outcome was considered to be successful as described in the methods section (see *postoperative measurements*). In this study, a successful outcome was achieved in 41 (74.5%) of 55 patients, while 13 (23.6%) 55 of patients were under-corrected, and 1 (1.8%) of 55 patients were overcorrected at 3 years after surgery. In the True CI group, 30 (85.7%) 35 of patients showed surgical success, and 5 (14.3%) of 35 patients had recurrences. In the Masked CI group, 11 (55%) of 20 patients demonstrated surgical success, 8 (40%) of 20 patients had recurrences, and 1 (5%) of 20 patients was overcorrected. There was a statistically significant difference in surgical outcomes between the two groups (p = 0.028). None of the patients showed postoperative amblyopia owing to consecutive esotropia, persistent diplopia, torsional effect or A-V pattern misalignment, or limitation of abduction.

**Figure 1:**
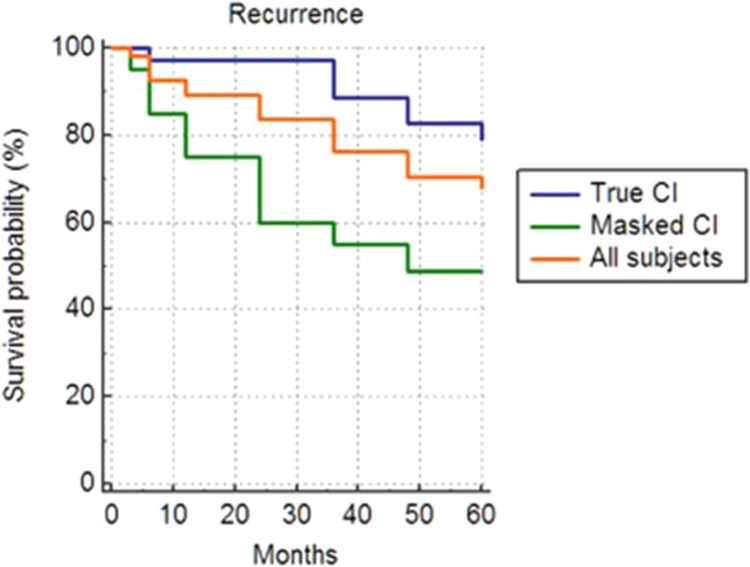
Kaplan-Meier survival plots and cumulative probabilities of success at 3 postoperative years according to life-time analysis of all the subjects in this study as well as of the two groups of intermittent exotropia with convergence insufficiency according to their response to diagnostic monocular occlusion for 2 hours. The overall cumulative probability of surgical success at 3 postoperative years was 76%. Mean recurrence was 49.9 ± 2.5 months (95% confidence interval, 44.980–54.737) in all the subjects with intermittent exotropia with convergence insufficiency. Cumulative probabilities of surgical success at 3 postoperative years were 89% in the True-CI group and 55% in the Masked-CI group. Mean recurrence in the True CI group was 55.7 ± 2.0 months (95% confidence interval, 51.725–59.658), and that in the Masked CI group was 39.6 ± 5.0 months (95% confidence interval, 29.790–49.443). There was a statistically significant difference in survival curves between the two groups. (p = 0.0052, Log-rank test).

According to Kaplan-Meier survival analysis, cumulative probabilities of surgical success at 3 years postoperatively were 76%, and the mean recurrence was 49.9 ± 2.5 months (95% confidence interval, 44.980–54.737) in all the patients of this study. The True CI and Masked CI groups revealed cumulative success rates of 89% and 55%, respectively. Mean recurrence in the True CI group was 55.7 ± 2.0 months (95% confidence interval, 51.725–59.658), whereas that in the Masked CI group was 39.6 ± 5.0 months (95% confidence interval, 29.790–49.443) in the Masked CI group. A statistically significant difference in the mean recurrence was observed between the two groups (p = 0.0052, Log-rank test) (Fig. 1).

## Discussion

The present study demonstrated that slanted bilateral LR recession in 55 children with IXT and CI successfully reduced both distance and near exodeviations to 3.5 PD and 4.4 PD, respectively, and collapsed near-distance differences from 10.9 PD to 1.0 PD at 3 years after surgery. The impact of slanted LR recession on the collapse of near-distance differences was 8.8 PD/mm. The cumulative probabilities of surgical success were 76% at 3 years after surgery, and the mean recurrence was 50 months.

This surgical outcome may be the result of slanted LR recession, which had first been introduced by Snir *et al*.^1^ based on the findings from Scott’s study^17^. Snir *et al*.^1^ reported that 11 of 12 patients had residual exodeviations of <8 PD, and that their mean near-distance difference decreased from 14 PD to 2.9 PD, with a 4.6 PD/mm reduction in near-distance difference. Our previous study reported that surgical success was achieved in 84% of children with IXT and CI, and that the slanting procedure reduced the near-distance difference by 8.7 PD/mm at 6 months postoperatively^15^. Kwon and Lee^16^ reported that the 12-month postoperative success rate of the slanting procedure was 50.9%, and that this technique caused a 4.6 PD/mm reduction in the near-distance difference. Our success rate of 74.5% is relatively superior to the success rates of other surgical procedures for IXT with CI, which range between 18% and 67%^2–4,7–10,12^. Our recurrence rate was 24% and the recurrence rates of other surgical procedures for IXT with CI ranged between 8% and 100%^7–10,12,14^. Two previous studies reported success rates ranging between 84% and 92% for slanted LR recession, however, those studies had a small sample size (12 and 31 patients) and relatively short follow-up periods (6 and 12 months)^1,15^. The strength of our study is that its results are based on a large number (55) of patients with a longer follow-up period (53 ± 12.5 months) as opposed to those employed in previous studies. In this study, the impact of slanting LR recession on the collapse of the near-distance difference was 8.8 PD/mm, which is almost 2-fold higher than 4.6 PD/mm reported in previous studies^1,16^. We only included children with IXT and CI who had no prior treatment (whether surgical or non-surgical).

Nevertheless, our results are inconsistent with those reported by Bothun^18^ and Kushner^19^. They suggested that recession alone was primarily responsible for surgical outcomes regardless of slanting. Kushner^19^ inferred that the edge tension difference after slanting procedure may be equalized and the effect of slanting is neutralized within weeks by muscular salcomere remodelings. In addition, Archer^20^ reported that a larger preoperative distance-near incomitance was associated with a greater reduction in distance-near incomitance with surgery, irrespective of which muscles undergo surgery. This study supports the concept that surgery apparently acts to normalize the preoperative distance-near incomitance^20^. However, the successful collapse of near-distance differences after slanted LR recessions was well maintained for more than 3 postoperative years in this study.

One unexpected and noteworthy finding in this study was that patients in the True CI group demonstrated surgical outcomes superior to those demonstrated by patients in the Masked CI group after slanted LR recession. We speculate this inter-group difference in surgical outcomes may be somewhat related to the change in fusional convergence after slanting procedures. In the Masked CI group, the true exodeviation at near has been masked by fusional convergence, i.e., accommodative convergence or proximal convergence^13^. Collapsed near-distance difference after slanted LR recession may decrease the necessity of using fusional convergence, which had previously been beneficial for IXT patients with Masked CI owing to the compensation of their preoperative greater exodeviation at near. This postoperative decrease in fusional convergence may induce a postoperative exo-drift at near and then lead to relatively earlier recurrence in the Masked CI group compared with the True CI group (40 versus 56 months).

There are certain limitations in the present study. First, children included in this study had relatively smaller near-distance differences of 10.9 PD than those of patients in other studies (11.3 PD - 14.5 PD)^1,2,7–9^. We postulate that these relatively smaller near-distance differences may be one of the reasons for our superior surgical outcomes of IXT with CI. Second, measurements of the AC/A ratio or degree of accommodative amplitudes were not available in most of the patients. This information might help to explain the reason for worse surgical outcomes in the patients of the Masked CI group compared with those of the True CI group. Third, the fusional amplitudes and near point of convergence (NPC) were not evaluated in this cohort; for IXT with CI is known as having a normal NPC and fusional amplitudes^21^. Finally, we did not preoperatively measure the degree of slant of the original LR muscle insertion during strabismus surgery. There may be a small amount of difference between the shortest distances from the limbus to the upper pole of the LR muscle and that from the limbus to the lower pole of the LR muscle before surgery; however, it would have had little impact on our findings of this study.

In conclusion, slanted bilateral LR recession performed in children with IXT and CI successfully reduced the distance and near exodeviations and collapsed near-distance differences over 3 postoperative years. Particularly, patients in the True CI group (having near-distance differences ≥10 PD maintained after monocular occlusion) demonstrated surgical outcomes superior to those demonstrated by patients in the Masked CI group (having near-distance differences <10 PD, and ≥10 PD prior to and after monocular occlusion). Further study will be necessary in planning the best surgical procedure for patients with IXT having near-distance differences <10 PD and ≥10 PD prior to and after monocular occlusion for 2 hours.

## Materials and Methods

This prospective study included 55 patients having IXT with CI who underwent slanted bilateral LR recession between December 2012 and April 2015. On the day before strabismus surgery, patients underwent monocular occlusion for 2 hours prior to examination. All patients had a greater exodeviation at near fixation than at distance by ≥ 10 PD after preoperative monocular occlusion. Their minimum postoperative follow-up period was 36 months, except for patients who required reoperation after the primary surgery. Patients with a history of ocular or strabismus surgery; concomitant ophthalmological problems including amblyopia; presence of vertical, torsional, or A-V pattern of strabismus; simultaneous vertical or oblique muscle surgery; other systemic disorders including neurologic diseases; or a postoperative follow-up duration of less than 36 months were excluded from this study. Preoperatively, no patients underwent occlusion therapy or myopic overcorrection, nor were they prescribed prism glasses. This study was approved by the Institutional Review Board of Kyungpook National University Hospital and was conducted in accordance with the tenets of the Declaration of Helsinki. All patients or their parents, in case of children, provided their informed consent.

### Preoperative ophthalmologic examinations

All patients underwent thorough ophthalmologic examinations, including measurements of best-corrected visual acuity and refractive errors, slit-lamp examination, and fundus photographs. We measured deviation angles at near and distance fixations by performing alternate prism cover test using accommodative targets with best optical correction. For patients with hyperopia, glasses providing a partial correction, namely 1.0 to 1.5 diopters (D) lesser than the full cycloplegic refraction were prescribed. Additional measurements were performed after preoperative monocular occlusion of the non-dominant eye for 2 hours, and post-occlusion measurements were taken before allowing the patient to regain binocular fusion. Abnormalities in duction and version and the presence of A-V pattern were checked. Torsion was measured based on fundus photographs. A binocular test for fusion and stereopsis was performed for all patients. Stereopsis was evaluated by the Titmus test at near, and fusion was evaluated by the Worth four-dot test at distance and near. Refractive errors were measured by cycloplegic refraction and recorded as spherical equivalent values. Data collection included patient’s age at surgery, sex, preoperative and postoperative deviation measurements at distance and near fixation, and the amount of near-distance differences.

### Subgroup classification by response to diagnostic monocular occlusion

Patients were divided into two groups according to their response to monocular occlusion performed for 2 hours prior to strabismus surgery^13^. The True CI group was defined as having near-distance differences of ≥10 PD prior to and after occlusion. The Masked CI group was defined as having near-distance differences of <10 PD and ≥10 PD prior to and after occlusion, respectively, although patients in this group were initially considered to have a basic-type IXT, their exodeviation at near fixation increased after preoperative monocular occlusion.

### Surgical procedures

All surgeries were performed under general anesthesia by the same surgeon (BYC). The upper pole of the LR muscle was recessed according to the distance deviation angle, and the lower pole of the LR muscle was recessed according to the near deviation angle, creating a new and oblique insertion in comparison with the original insertion^1^. As the deviation was greater at near than at distance, the lower pole was recessed more extensively than the upper pole (from 1.0 to 2.0 mm)^1^. Surgery was based on the largest deviation which measured after preoperative monocular occlusion for 2 hours at both near and distance fixation.

### Postoperative measurements

Measurements of postoperative alignment at distance and near in the primary position were done at 1 week, at 1, 3, 6, 12, 24, 36 months; and during their last visit. Surgical outcome was considered successful if patients had a distant deviation in the primary position between ≤8 PD of exodeviation and ≤5 PD of esodeviation as well as a near-distance difference of ≤8 PD. Recurrence was defined as>8 PD of exodeviation, and overcorrection was defined as>5 PD of esodeviaton. Reoperation for overcorrection was performed if esotropia of>20 PD persisted or increased for 6 months postoperatively. The impact of slanted LR recession on the reduction of the near-distance difference was calculated by dividing the mean reduction in near-distance difference after surgery by the mean amount of slanted LR recession, which was defined as the mean difference between the new upper and lower poles of the slanted LR in comparison with their original insertions^1^. Main outcome measurements included cumulative probabilities of success at 3 years after surgery and the mean recurrence. Inter-group analysis was performed according to the response to preoperative monocular occlusion for 2 hours.

### Statistical analysis

Statistical analyses were performed using MedCalc software (version 19.0.7. MedCalc. Inc., Belgium) and SPSS software (version 18.0, SPSS, Inc., USA). Paired *t-*test was used to compare the preoperative and postoperative deviation angles at distance and near as well as the differences between them. Mann-Whitney U-test was used to compare the data between the two groups. The cumulative probabilities of success were assessed by conducing Kaplan-Meier survival analysis. Differences were considered significant if p-values were less than 0.05.
